# Impact of Minimal Incision Repair of Rectus Abdominis Diastasis on Quality of Life and Stress Incontinence: A Prospective Study

**DOI:** 10.3389/jaws.2024.13830

**Published:** 2025-02-04

**Authors:** Asmatullah Katawazai, Göran Wallin, Anna Ärlebäck, Gabriel Sandblom

**Affiliations:** ^1^ Departments of Surgery, Faculty of Medicine and Health, University Hospital Örebro, School of Medical Sciences, Örebro University, Örebro, Sweden; ^2^ Olaus Petri Healthcare Centre, Örebro, Sweden; ^3^ Department of Clinical Science and Education Södersjukhuset, Karolinska Institutet, Stockholm, Sweden; ^4^ Department of Surgery, Södersjukhuset, Stockholm, Sweden

**Keywords:** rectus abdominis diastasis, quality of life, ventral hernia, linea alba, postpartum rectus diastasis

## Abstract

**Aim:**

This study evaluates the impact of the minimal incision repair of rectus abdominis diastasis (MIRRAD) procedure on physical activity, muscle strength, quality of life, and overall satisfaction in women with postpartum rectus abdominis diastasis (PP-RAD).

**Methods:**

A cohort of 31 female patients, aged 20–50 years, diagnosed with PP-RAD unresponsive to conservative treatment, underwent the MIRRAD procedure. Assessments were conducted preoperatively and 1 year postoperatively, these included the Modified Abdominal Trunk Function Protocol (MATFP), Disability Rating Index (DRI), and Urinary Disability Index (UDI) questionnaires. Physical activity intensity was monitored using accelerometers.

**Results:**

Significant improvements were observed in vigorous physical activities (Z = −2.352, *p* = 0.019), vector magnitude counts per minute (Z = −2.163, *p* = 0.031), and steps per minute (Z = −3.131, *p* = 0.002). DRI showed significant improvements in physical tasks like dressing, walking, and strenuous work (Z ranging from −2.705 to −4.603, *p* < 0.001). UDI indicated significant improvements in urinary symptoms, including reduced frequency (Z = −2.984, *p* = 0.003) and less urinary leakage (Z = −2.357, *p* = 0.018). MATFP demonstrated gains in back and abdominal muscle strength (Z = −4.321, *p* < 0.001) and trunk stability (Z = −3.991, *p* < 0.001).

**Conclusion:**

The MIRRAD procedure significantly improves physical strength, trunk stability, and urinary function, enhancing daily activities and overall physical health in women with PP-RAD. Further research is recommended to evaluate long-term outcomes.

## Introduction

Rectus Abdominis Diastasis (RAD) is defined as separation of the rectus muscles by more than 2 cm [1]. It is a common condition where separation of the rectus abdominis muscles results in a widening of the linea alba, the connective tissue running down the midline of the abdomen. This separation is often due to increased intra-abdominal pressure and hormonal changes, which can arise for various reasons, most notably during pregnancy. The expanding uterus can stretch the abdominal wall, leading to RAD during pregnancy. This usually reverts, but in some women this leads to postpartum rectus abdominis diastasis (PP-RAD) [2].

PP-RAD can cause significant functional impairments and a range of symptoms. A primary concern is the instability and weakness of the abdominal wall resulting from compromised muscular and fascial integrity. This instability can weaken core strength and trunk function, potentially affecting the pelvic floor muscles. The condition manifests with both direct and indirect symptoms that can significantly impact daily life and physical activities.

Direct symptoms of PP-RAD include a visible bulge or “pooch” along the midline of the abdomen, especially noticeable during activities that engage the abdominal muscles such as sit-ups or lifting. This can lead to abdominal discomfort and a sense of weakness due to the lack of adequate core support. Research also indicates that PP-RAD can impair trunk biomechanics, triggering a series of musculoskeletal problems [3, 4].

Indirect symptoms linked to PP-RAD include low back pain, pelvic discomfort, and urinary stress incontinence. These occur because the abdominal and pelvic floor muscles provide less support, crucial for maintaining posture, spinal stability, and continence. Studies have shown that women with PP-RAD often report reduced quality of life due to these symptoms, experiencing difficulties in performing everyday tasks, participating in physical activities, or returning to pre-pregnancy exercise routines [1, 2, 5].

In addition to physical symptoms, PP-RAD can have substantial psychological effects. Many women report a negative body image and diminished self-confidence, as the persistent abdominal bulge can make them appear “pregnant,” causing social discomfort and emotional distress. Recent studies have linked these body image issues with lower self-esteem and a reluctance to engage in social or physical activities [6].

Complications associated with PP-RAD include an increased risk for hernias along the linea alba, particularly midline hernias where abdominal contents protrude through the weakened fascia. Additionally, PP-RAD can lead to hyperlordosis [7].

Recent research has advanced our understanding of RAD complications and management strategies. A 2021 systematic review published in the *Journal of Women’s Health Physical Therapy* highlighted the effectiveness of targeted physiotherapy interventions in managing PP-RAD symptoms [4]. These interventions focus on strengthening deep abdominal muscles and improving pelvic floor function, which can alleviate symptoms like low back pain and incontinence. Studies have shown that surgical correction of PP-RAD enhances functional outcomes such as core strength and pelvic stability. Women who opted for surgical repair reported higher satisfaction with their body image and fewer symptoms related to back pain and urinary incontinence compared to those who chose conservative management [8, 9].

While some women may find relief through physiotherapy and lifestyle modifications, others, particularly those with severe diastasis or associated hernias, may require surgical intervention. A comprehensive understanding of the wide range of symptoms and treatment options is crucial for providing holistic care that addresses both the physical and psychological impacts of PP-RAD [10, 11].

In conclusion, PP-RAD is more than a cosmetic concern; it has profound implications for core function, posture, and overall quality of life. Continued research is refining our understanding and management of this condition, offering hope for effective treatments that enhance both the physical and emotional wellbeing of these women.

The aim of this study was to evaluate the impact of a minimally incision repair of rectus abdominis diastasis procedure on quality of life and daily function.

## Materials and Methods

### Study Design and Participants

This prospective study included women diagnosed with postpartum rectus abdominis diastasis (PP-RAD) with an inter-rectus distance of ≥3 cm. The study was conducted at the Hernia Center, Region Örebro Län, Sweden, from October 2021 to August 2024. The study has been registered at ClinicalTrials.gov. All procedures were performed in compliance with the European Union’s General Data Protection Regulation (GDPR).

Inclusion Criteria:

• Female patients aged 20–50 years.
• Diagnosed with PP-RAD with an inter-rectus distance of ≥3 cm.
• At least 12 months postpartum and Unresponsive to conservative treatment.
• Decided against future pregnancies.
• No previous surgery involving the linea alba.

Exclusion Criteria:

• Inability or unwillingness to provide informed consent.
• Contraindications to general anesthesia.
• Pregnant or plan to become pregnant in the future.
• BMI >32 kg/m^2^

These patients, aged between 20 and 50 years, were all diagnosed with a minimum inter-rectus separation of 3 cm. After attempting conservative treatment without satisfactory improvement, they opted for surgical repair. To be eligible for the study, participants were required to be at least 1 year postpartum and to have decided against future pregnancies, as per the study criteria. All participants received comprehensive information about the study, both verbally and in writing.

**FIGURE 1:**
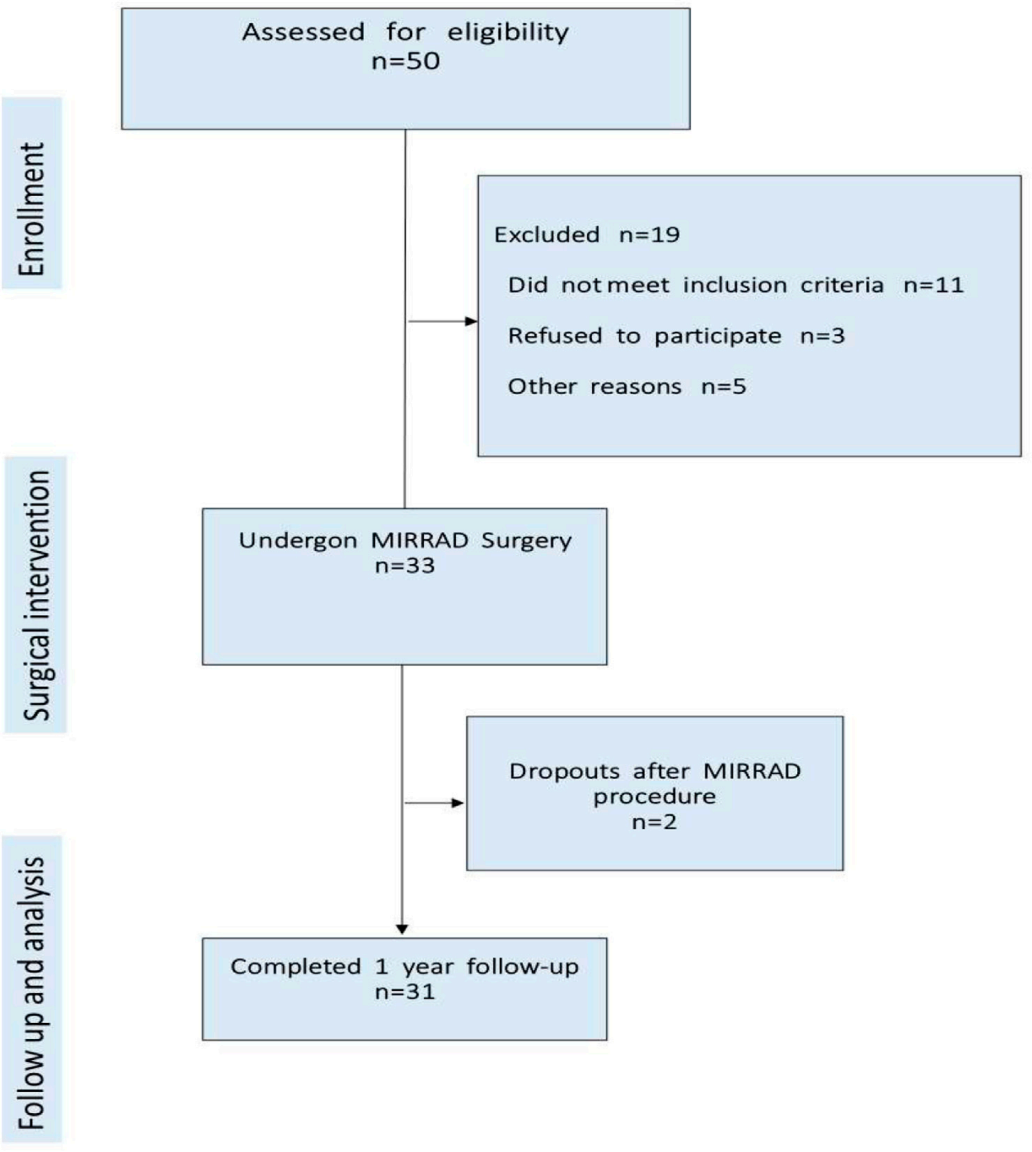
Patient election and follow-up.

A total of 50 patients were assessed for eligibility. Of these, 33 met the inclusion criteria and provided informed consent to participate in the study. A flowchart detailing patient selection, inclusion, exclusion, and follow-up is presented in Figure 1.

The primary outcomes assessed were improvements in physical health and overall life satisfaction.

### Reasons for Exclusion

Out of 50 eligible patients assessed, 19 were excluded prior to surgery for reasons including not meet inclusion criteria (11), refusal to participate (3), seeking repairs elsewhere (3) and undergoing surgery elsewhere (2). Consequently, 33 patients underwent MIRRAD procedure. Two patients were excluded post-surgery, resulting in a final analysis of 31 patients.

Most patients were referred to the Hernia Center due to a diagnosed hernia or suspected hernia in the linea alba. All patients had at least one hernia, with the majority having hernias measuring ≤1 cm. Additionally, all patients presented with an inter-rectus distance (D2 H1) of at least 3 cm, with a mean width of 4.4 cm (SD ± 1.0 cm) (Table 1).

**TABLE 1:** European Hernia Society (EHS) classification of RD guidelines.

T (Type)	D (inter-rectus distance)	H (concomitant umbilical and/or epigastric hernia)
T1 = after pregnancy	D1 = >2 cm	H0 = Without
	D2 = >3–5 cm	
T2 = With adiposity		H1 = Present
	D3 = >5 cm	

Each participant completed the Disability Rating Index (DRI), the Urogenital Distress Inventory-6 (UDI-6), and the Incontinence Impact Questionnaire-7 (IIQ-7) both before the surgery and 1 year postoperatively [12, 13]. Additionally, they underwent a Modified Abdominal Trunk Function Protocol (MATFP) assessment, conducted by a certified physiotherapist, both preoperatively and 1 year after the surgical repair [14]. Before surgery, participants were also provided with an accelerometer to monitor their physical activity levels.

### MIRRAD Procedure

**FIGURE 2:**
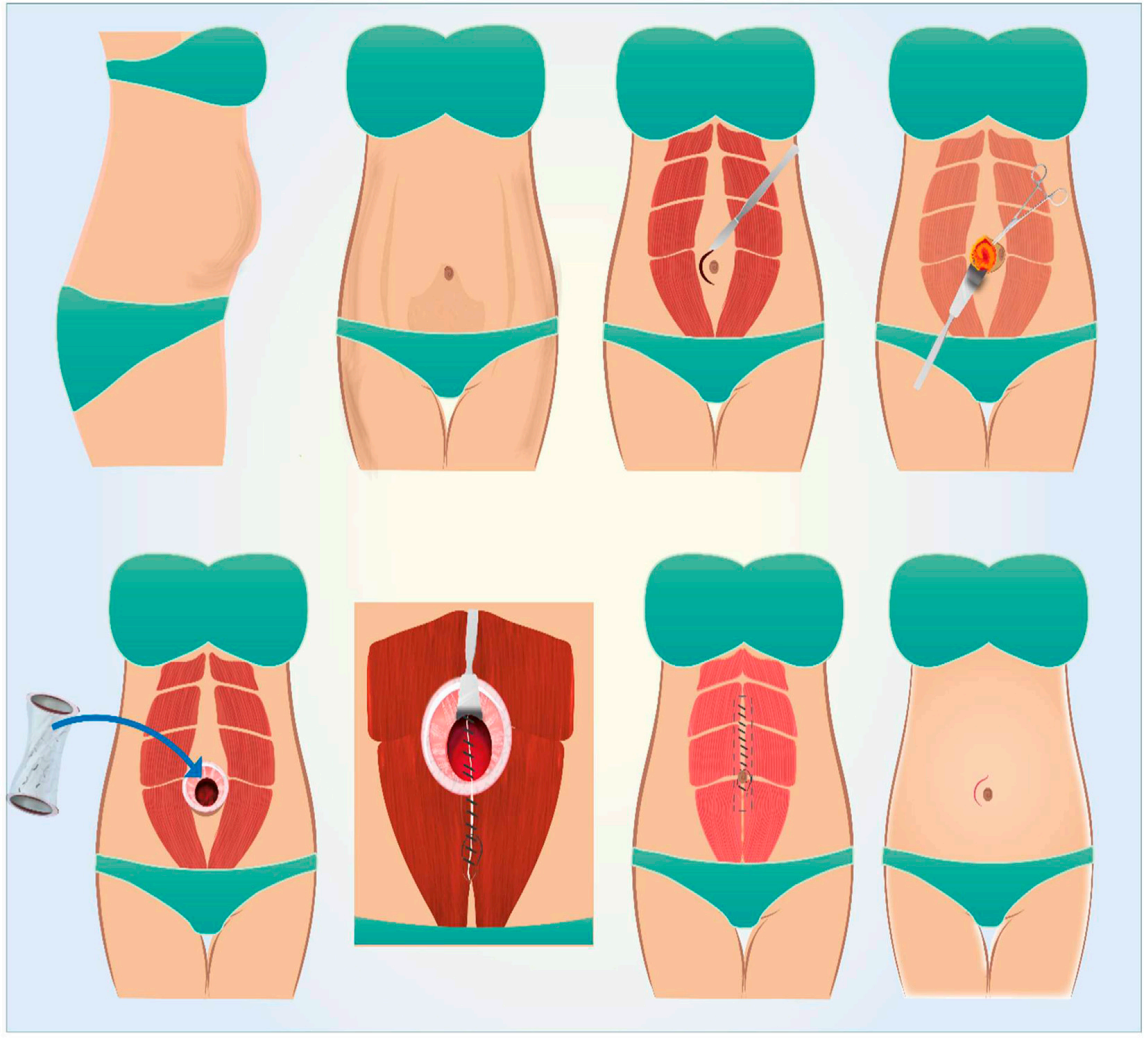
Illustrating the MIRRAD repair procedure. Illust. from the author’s library.

Minimally Incision Repair of Rectus Abdominis Diastasis (MIRRAD) surgery is a minimal incision procedure to plicate PP-RAD. The procedure is performed under general anaesthesia through a small skin incision near the umbilicus. It involves the plication of the rectus abdominis diastasis using barbed sutures as double-line suture, and if a concomitant hernia is found, it is sutured with 2.0 prolene before the plication. The umbilicus is then reattached to the fascia with single stitch of 4-0 PDS. The skin is closed with absorbable intradermal 3.0 Monocryl sutures, allowing for same-day discharge, which reduces hospital stay and cost Figure 2. All patients were provided an elastic abdominal binder before surgery and were advised to use it 6 weeks postoperatively. The hernia defects were repaired using non-absorbable sutures, while the rectus diastasis was closed with a 0-PDS double-lined barbed suture. All procedures were performed by the same experienced consultant surgeon at the Hernia Center, Region Örebro Län, Sweden. This repair method utilized an open surgical approach through a small skin incision near the umbilicus. While achieving similar results to the SCOLA method, the MIRRAD technique does not require mesh reinforcement. This method may serve as a bridge between traditional open surgeries and advanced laparoscopic techniques, offering a cost-effective and efficient solution for abdominal wall reconstruction. This technique is particularly appealing to patients seeking a less invasive option, delivering a balance between effective repair and a faster recovery.

### Data Collection and Analysis

All women included in the study were aged between 20 and 50 years, with a BMI range of 18.8–30.1. None of the participants had undergone previous surgery involving the linea alba. According to the study protocol, all women were enrolled at least 12 months after their most recent delivery and had decided not to pursue further pregnancies. Current illnesses (classified by ICD codes) and ongoing medications were recorded for all participants.

Data were collected electronically using Greenlight Guru Clinical (formerly Smart-trial) software. All data was stored according to the European data protection policy. Standardized assessments were conducted preoperatively and at 1 year postoperatively. An accelerometer is a device that measures acceleration forces in one or more directions, typically in three axes: X (horizontal), Y (vertical), and Z (depth). It detects changes in velocity over time, which can be used to measure motion, orientation, and vibration. It works by sensing motion through tiny sensors called MEMS (Micro-Electro-Mechanical Systems). These parts move when the device moves, generating an electrical signal that the device uses to calculate acceleration. The participants carried the device at all times while awake (except during showers and baths) for 5 days. After this period, they returned the device in a padded envelope to the principal researcher for computerised analysis. Each participant also kept a simple activity diary during this period with the accelerometer.

#### Physical Function and Strength Assessments

Modified Abdominal Trunk Function Protocol (MATFP): Conducted by a certified physiotherapist to evaluate abdominal wall strength and trunk stability.

Accelerometer Monitoring: Physical activity levels were measured using ActiGraph wGT3X-BT accelerometers over five consecutive days. Participants were instructed to wear the device during waking hours, except during bathing or swimming.

#### Questionnaires

Disability Rating Index (DRI): Assessed the impact of symptoms on daily activities.

Urogenital Distress Inventory-6 (UDI-6): Evaluated urinary symptoms.

Incontinence Impact Questionnaire-7 (IIQ-7): Measured the impact of urinary incontinence on quality of life.

Pain Assessment: Visual Analogue Scale (VAS): Used to measure postoperative pain at 4 h, 1 week, and 1 month after surgery.

### Statistical Analysis

Data were exported to IBM SPSS Statistics version 29.0.0.0 for analysis. Continuous variables were expressed as mean ± standard deviation (SD) or median with interquartile range (IQR) as appropriate. The Wilcoxon signed-rank test was used to compare preoperative and postoperative outcomes. A *p*-value of <0.05 was considered statistically significant.

### Ethical Considerations

The study was conducted in accordance with the Declaration of Helsinki and complied with the GDPR for data protection. All participants provided written informed consent after receiving detailed information about the study’s purpose, procedures, and potential risks and benefits. The study was approved (Dnr. 2021-04599) by the Swedish Board of Ethics (Etikprövningsmyndigheten).

### Recovery and Follow-Up

All study patients were followed up clinically by a surgeon and a physiotherapist 1 year after the procedure. Patients were encouraged to contact the clinic if they experienced any signs of complications. If any complications were suspected, the patient was invited for an additional visit, which included clinical examination, laboratory tests, or radiological assessment as necessary. All events related to the procedure were documented in Smart-trial, and additional visits were scheduled according to local protocols. Additionally, all patients were provided with an abdominal binder and advised to use it for 6 weeks postoperatively.

## Results

### Patient Enrollment and Follow-Up

Out of 50 patients assessed, 33 met the inclusion criteria and were enrolled in the study. Two patients did not participate in the 1-year follow-up. One cited the long distance to the hospital, and the other provided no explanation. Both reported no complications at the 1-month postoperative follow-up. One-year follow-up data were successfully collected from the 31 participants who completed the study (Figure 1). Mean preoperative inter-rectus distance was 4.4 ± 1.0 cm. All participants were at least 1 year postpartum and had decided against future pregnancies. At the 1-year follow-up, no cases of hernia or rectus diastasis recurrence were reported. Two patients developed seromas, confirmed radiologically at 1 week and 1-month postoperative. Nausea was reported by five patients. No other surgery-related complications were observed. Postoperative pain was assessed using a Visual Analogue Scale (VAS), with mean pain scores of 2.8 at 4 h, 3.2 at 1 week, and 1.7 at 1 month following surgery.

The Wilcoxon signed rank test showed statistically significant improvements in physical activities, strength, and overall functional ability 1 year postoperatively compared to preoperative assessments. The analysis demonstrated significant enhancements in physical activities, strength, and overall ability. Notable reductions were recorded in urinary frequency, urinary leakage, and pain, alongside improvements in the ability to engage in leisure activities.

However, trends in household chores, travelling, and social activities did not reach statistical significance.

### Accelerometer

Data were collected pre- and postoperatively to assess changes in physical activity. A Wilcoxon signed rank test revealed significant improvements in vigorous activities (Z = −2.352, *p* = 0.019), vector magnitude counts per minute (Z = −2.163, *p* = 0.031), and steps per minute (Z = −3.131, *p* = 0.002), indicating enhanced physical performance following surgery (Table 2).

**TABLE 2:** Accelerometer data summary using count per minute (CPM).

Type of physical activity (preoperative vs. postoperative)	Z	*P*-value
Light physical activity - preoperative vs. postoperative	−1.207	0.227
Moderate physical activity - preoperative vs. postoperative	−1.731	0.083
Vigorous physical activity - preoperative vs. postoperative	−2.352	0.019
Vector_Magnitude_CPM	−2.163	0.031
Steps Per Minute - preoperative vs. postoperative	−3.131	0.002

Wilcoxon Signed Rank Test. Based on positive rank.

Source: Katawazai et al., 2025.

### Disability Rating Index (DRI)

The postoperative data showed significant improvements across all assessed activities. Dressing and undressing without assistance showed a statistically significant improvement (*p* = 0.048). Walking, climbing stairs, sitting for an extended period, standing bent over a sink, carrying a bag or package, making a bed, running, performing light physical work, performing strenuous physical work, and engaging in exercise or sports all demonstrated highly significant improvements, with Z values ranging from −2.705 to −4.603 and (*p*- < 0.001). Overall, the results show considerable improvements in physical abilities and daily activities 1 year after the procedure (Table 3).

**TABLE 3:** DRI activity improvements pre- and 1-year post-operative.

Type of activity (Preoperative vs. postoperative)	Z	P-value
Dressing and undressing without assistance – Preop. Vs. postop.	−1.974^a^	<0.048
Walking – Preop. Vs. postop.	−3.466^a^	<0.001
Climbing stairs – Preop. Vs. postop.	−3.450^a^	<0.001
Sitting for an extended period – Preop. Vs. postop.	−3.798^a^	<0.001
Standing bent over a sink – Preop. Vs. postop.	−3.357^a^	<0.001
Carrying a bag or package – Preop. Vs. postop.	−4.023^a^	<0.001
Making a bed – Preop. Vs. postop.	−2.705^a^	<0.001
Running – Preop. Vs. postop.	−3.933^a^	<0.001
Physical work – Preop. Vs. postop.	−3.836^a^	<0.001
Strenuous physical work – Preop. Vs. postop.	−4.603^a^	<0.001
Exercise/sports – Preop. Vs. postop.	−4.389^a^	<0.001

Wilcoxon Signed Rank Test.

^a^ Based on positive rank.

Source: Katawazai et al., 2025.

### Urogenital Distress Inventory-6, Incontinence Impact Questionnaire−7 (UDI-6, IIQ-7)

The postoperative results show significant improvements in several areas. There was a notable reduction in the frequency of urination and in urinary urgency incontinence associated with physical activity, coughing, or sneezing. Minor leakage incidents also decreased significantly. Additionally, improvements were observed in difficulty emptying the bladder and in pain or discomfort in the lower abdomen or genital area.

Significant improvements were also seen in leisure activities and mental health, with a reduction in frustration levels. There were trends towards improvement in performing household chores, travelling, and participating in social activities, although these were not statistically significant (Table 4).

**TABLE 4:** UDI-6 and IIQ-7 results pre- and 1-year post-operation.

Type of activity/Symptom (Preoperative vs. postoperative)	Z	P-value
Need to go to the bathroom very often – Preop. Vs. postop.	−2.984^a^	0.003
Urinary leakage associated with a strong need to urinate – Preop. Vs. postop.	−2.357^a^	0.018
Urinary leakage associated with physical activity, coughing, or sneezing – Preop. Vs. postop.	−2.289^a^	0.022
Small amounts of urinary leakage (drops) – Preop. Vs. postop.	−3.000^a^	0.003
Postop._Difficulty emptying the bladder – Preop. Vs. postop.	−2.352^a^	0.019
Pain or discomfort in the lower abdomen or genital area – Preop. Vs. postop.	−2.951^a^	0.003
Postop._Ability to perform household chores (cooking, cleaning, laundry) – Preop. Vs. postop.	−1.732^a^	0.083
Postop._Leisure activities such as walking, swimming, or other physical activities – Preop. Vs. postop.	−3.957^a^	<0.001
Entertainment (e.g., movies, concerts) – Preop. Vs. postop.	−0.378^a^	0.705
Ability to travel by car or bus more than 30 min from home – Preop. Vs. postop.	−1.890^a^	0.059
Participation in social activities outside the home – Preop. Vs. postop.	−1.811^a^	0.070
Mental health (nervousness, depression, etc.) – Preop. Vs. postop.	−2.887^a^	0.004
I feel prevented from doing what I want – Preop. Vs. postop.	−3.106^a^	0.002

Wilcoxon Signed Rank Test.

^a^ Based on negative rank.

Source: Katawazai et al., 2025.

### Modified Abdominal Trunk Function Protocol Assessment (MATFP)

The postoperative results showed significant improvements in muscle strength and trunk stability compared to the preoperative state. Both back and abdominal muscle strength increased significantly after the procedure, as did trunk stability in the side plank exercise. There were also notable improvements in overall levels reached in the assessment. Overall, these results indicate considerable gains in muscle strength and trunk stability following the procedure (Table 5).

**TABLE 5:** MATFP results pre- and 1-year post-operation.

MATFP results (preoperative vs. postoperative)	Z	P-value
Back muscle strength (number of seconds) – Preop. Vs. postop.	−4.206^a^	<0.001
Abdominal muscle strength (number of seconds) – Preop. Vs. postop.	−4.321^a^	<0.001
Trunk stability in side-plank (Side plank) (seconds) – Preop. Vs. postop.	−3.991^a^	<0.001
Reached level – Preop. Vs. postop.	−3.328^a^	<0.001
Lifting right leg – Preop. Vs. postop.	−2.646^a^	0.008
Lifting left leg – Preop. Vs. postop.	−1.265^a^	0.206

Wilcoxon Signed Rank Test.

^a^ Based on positive rank.

Source: Katawazai et al., 2025.

### Patient Satisfaction

All patients rated their satisfaction with their operation on a scale from 0 to 10 (out of 10), with a mean score of 8.65, indicating high satisfaction. Postoperative pain and discomfort levels were rated from 1 to 6 (out of 10), with an average of 2.32, suggesting low to moderate discomfort after surgery (Table 6).

**TABLE 6:** Patient satisfaction and discomfort scores.

	N	Minimum	Maximum	Mean	Std. Deviation
How satisfied is the patient with the operation?	31	2	10	8.6	1.7
How much discomfort has the patient experienced after the diastasis surgery?	31	1	6	2.3	1.3

Source: Katawazai et al., 2025.

Regarding abdominal wall stability 1-year post-surgery, 83.9% of patients reported feeling either quite better or much better, while only one patient reported no difference.

In summary, surgery resulted in high patient satisfaction, low postoperative pain and discomfort, and significant improvements in abdominal wall stability.

## Discussion

### Summary of Findings

In conclusion, the MIRRAD procedure significantly improves physical strength, trunk stability, and urinary function in women with PP-RAD. One year postoperatively, participants showed significant enhancements in physical activities, reduced urinary symptoms, and improved quality of life.

### Physical Function and Strength Improvements

The significant gains in back and abdominal muscle strength and trunk stability indicate the effectiveness of the MIRRAD procedure in restoring core muscle function. These improvements facilitated better performance in daily activities such as dressing, climbing stairs, and engaging in exercise, aligning with previous studies that highlight the benefits of surgical intervention for PP-RAD.

### Urinary and Pain-Related Outcomes

The significant reduction in urinary frequency, urgency, and leakage suggests that repairing the rectus diastasis positively impacts pelvic floor function. Improvements in pain and discomfort, as well as better leisure activities and mental health, highlight the comprehensive benefits of MIRRAD procedure beyond mere physical strength.

### Limitations and Strengths

This study has some limitations, the relatively small sample size of 31 patients may limit the generalizability of the findings. The lack of a control group prevents definitive conclusions about the procedure’s efficacy compared to non-surgical interventions. A 1-year follow-up may not capture long-term outcomes and potential late-onset complications. Despite these constraints, the study’s strengths include its prospective design and the use of validated outcome measures, which we believe add valuable insights into the treatment of rectus diastasis.

Future studies should include larger cohorts, control groups, and extended follow-up periods to validate these findings and assess the long-term effectiveness and safety of the MIRRAD procedure.

### Conclusion

The MIRRAD surgical procedure is effective in improving physical strength, trunk stability, and urinary function in women with PP-RAD. The technique offers a minimally invasive option with minimal complications and significant improvements in quality of life. Despite the study’s limitations, the findings contribute valuable insights and support the use of MIRRAD as a viable surgical intervention for PP-RAD.

## Data Availability

The original contributions presented in the study are included in the article/Supplementary Material, further inquiries can be directed to the corresponding author.

## Abbreviations

MIRRAD, Minimal Incision Repair of Rectus Abdominis Diastasis; RAD, Rectus Abdominis Diastasis; PP-RAD, Postpartum Rectus Abdominis Diastasis; MATFP, Modified Abdominal Trunk Function Protocol; VAS, Visual Analogue Scale; DRI, Disability Rating Index; UDI-6, IIQ-7, Urogenital Distress Inventory-6, Incontinence Impact Questionnaire −7; VHPQ, Ventral Hernia Pain Questionnaire.
