# Supplementary material for: Impact of Minimal Incision Repair of Rectus Abdominis Diastasis on Quality of Life and Stress Incontinence: A Prospective Study
Source: J Abdom Wall Surg. 2025 Feb 4;3:13830. doi: 10.3389/jaws.2024.13830 (PMC11833112; doi:10.3389/jaws.2024.13830)
Supplement: Supplementary file 1 [file DataSheet2.PDF]

Modified Abdominal Trunk Function Protocol

The tests are terminated if the patient can no longer continue or if they cannot maintain the specified important technical points for each test. A technical warning is issued; if the patient can correct their technique, the test continues until they can no longer continue or if the technique cannot be maintained once more (the same technical error is repeated once more).

1. Back Muscle Strength

Equipment: Stopwatch, carpet

The patient lies on their stomach on the floor with arms at their sides, hands at shoulder level, and elbows close to the body's sides.

The patient is instructed to slightly draw in the abdomen (pull the navel toward the spine), lift the upper body from the floor, gaze slightly forward, and maintain this horizontal position for as long as possible, up to a maximum of 240 seconds.

If the patient cannot maintain the position for the full time, the therapist/trainer stops the timing when the patient loses the position.

Technical points to observe

- The body is kept stable and in the same plane; no rotation occurs in any plane.
- The abdomen maintains a neutral position, i.e., no extra doming/coning, chest gripping, or pushing out the abdomen.

The test is terminated when any of these technical points cannot be maintained despite a warning. The test is terminated after a warning and when the compensation is performed once more.

Number of seconds: _________________

Was the test terminated due to pain or discomfort? Yes ☐ No ☐

If yes, indicate the location of the pain/discomfort: ______________________________________

2. Abdominal Muscle Strength

Equipment: Stopwatch, carpet

The patient lies on their back with arms crossed in front of the chest (hand on opposite shoulder) and with hips and knees bent, feet on the floor hip-width apart.

The patient is instructed to nod (tuck in the head), continue to lift the head and shoulders until the lower edge of the scapula (inferior angle) leaves the surface.

The patient is asked to maintain this position for as long as possible, up to a maximum of 240 seconds.

If the patient cannot maintain the position for the full time, the therapist/trainer stops the timing when the patient loses the position.

Technical points to observe

- The abdomen maintains a neutral position, i.e., no extra doming/coning.
- The lower edge of the scapula is off the floor/bench.

The test is terminated when any of these technical points cannot be maintained despite a warning. The test is terminated after a warning and when the compensation is performed once more.

Number of seconds: ____________________

Was the test terminated due to pain or discomfort? Yes ☐ No ☐

If yes, indicate the location of the pain/discomfort: ___________________________________

3. Core Stability in Side-Lying Position (Side Plank) Right on Knee

Equipment: Stopwatch, carpet

The patient lies on their side with the body straight and knees bent. With support from the forearm, the straightened body is slowly lifted from the floor to a position where only the lower leg/foot and lower forearm are in contact with the floor. In the final position, the body is kept straight in both the frontal and sagittal planes. It is progressed in three steps:

Step 2: Extend the upper arm toward the ceiling.

Step 3: Lift the upper leg up approximately 45 degrees toward the upper body.

If the patient cannot maintain the position for the full time, the therapist/trainer stops the timing when the patient loses the position.

Technical points to observe

The body is kept stable and in the same plane; no rotation occurs in any plane.

The back maintains a neutral position with a slight neutral curvature, i.e., no extra kyphosis or posterior pelvic tilt.

The abdomen maintains a neutral position, i.e., no extra doming/coning, chest gripping.

The test is terminated when any of these technical points cannot be maintained despite a warning. The test is terminated after a warning and when the compensation is performed once more.

Number of seconds: ____________________

Was the test terminated due to pain or discomfort? Yes ☐ No ☐

If yes, indicate the location of the pain/discomfort: ___________________________________

Core Stability in Side-Lying Position (Side Plank) Left on Knee

Equipment: Stopwatch, carpet<br><br>The patient lies on their side with the body straight and knees bent. With support from the forearm, the straightened body is slowly lifted from the floor to a position where only the lower leg/foot and lower forearm are in contact with the floor. In the final position, the body is held straight in both the frontal and sagittal planes. It is progressed in three steps:<br><br>Step 2: Extend the upper arm upward toward the ceiling.<br><br>Step 3: Also lift the upper leg up at approximately a 45-degree angle toward the upper body.<br><br>If the patient cannot maintain the position for the full duration, the therapist/trainer stops the timing when the patient loses the position.

Technical points to observe

- The body is kept stable and in the same plane; no abnormal rotation occurs in any plane.
- The back maintains a neutral position with a slight neutral curvature, i.e., no extra kyphosis or posterior pelvic tilt.
- The abdomen maintains a neutral position, i.e., no extra doming/coning, chest gripping.

The test is terminated when any of these technical points cannot be maintained despite a warning.

Number of seconds: ____________________

Was the test terminated due to pain or discomfort? Yes ☐ No ☐

If yes, indicate the location of the pain/discomfort: ___________________________________

4. Core Stability in Prone Support on Elbows (Core Muscle Strength and Stability Test)

Equipment: Stopwatch, carpet

The patient lies on their stomach, bends the knees, and places the forearms close to the body, ready to come up into a plank on knees. The patient lightly activates the pelvic floor and abdomen (draws together and pulls the abdomen in slightly for more support) and pushes up into a plank on knees where only the forearms and knees support against the floor.

Level 1: Hold the basic position for 60 seconds.

Level 2: Straighten the knees and come up onto the toes. Hold for 60 seconds.

Level 3: Lift the right arm from the floor and extend it straight forward in line with the back, neck, and head. Hold for 15 seconds.

Level 4: Switch to the left arm. Hold for 15 seconds.

Level 5: Lift the right leg from the floor until the right toe is level with the left heel. Hold for 15 seconds.

Level 6: Switch to the left leg.

Level 7: Return the left leg to the basic position, then lift the left leg and right arm. Hold for 15 seconds.

Level 8: Lift the right leg and left arm. Hold for 15 seconds. Return to the basic position and hold for 30 seconds to conclude the test.

If the patient cannot maintain the position for the full time, the therapist/trainer stops the timing when the patient loses the position.

Technical points to observe

- The body is kept stable and in the same plane; no abnormal rotation occurs in any plane.
- The back maintains a neutral position with a slight neutral curvature, i.e., no extra kyphosis or posterior pelvic tilt.
- The abdomen maintains a neutral position, i.e., no extra doming/coning, chest gripping.

The test is terminated when any of these technical points cannot be maintained despite a warning. The test is terminated after a warning and when the compensation is performed once more.

Level achieved: ____________________

Number of seconds: ____________________

Was the test terminated due to pain or discomfort? Yes ☐ No ☐

If yes, indicate the location of the pain/discomfort: ________________________________

5. Active Straight Leg Raising (ASLR)

The patient lies on their back with straight legs and a 20 cm distance between the feet.<br><br>Inform the patient that a straight leg lift will be performed and that they should note any difficulty in lifting the leg or if it provokes pain in the back or pelvic region.<br><br>The patient is asked to lift one leg at a time off the surface with a straight knee approximately 20 cm and hold for a few seconds.

The therapist asks the patient:

a) "Did you experience any difficulty in lifting your right or left leg?"

Right leg:

1 ☐	2 ☐	3 ☐	4 ☐	5 ☐
Not difficulty	Minimal difficulty	Some difficulty	Difficult	Impossible to perform

Left leg:

1 ☐	2 ☐	3 ☐	4 ☐	5 ☐
Not difficulty	Minimal difficulty	Some difficulty	Difficult	Impossible to perform

b) "Did you experience any pain in the back or around the pelvis when you lifted?"

Right leg: Yes ☐ No ☐

Left leg: Yes ☐ No ☐

c) The therapist assesses:

Was there a pelvic tilting in the horizontal plane on the opposite side of the lifted leg?

Pelvic tilting when lifting right leg: Yes ☐ No ☐

Pelvic tilting when lifting left leg: Yes ☐ No ☐

6. ASLR Endurance Test

The patient lies on their back with straight legs and a 20 cm distance between the feet. A metronome is used with the tempo set to 60 beats per minute (bpm).<br><br>Inform the patient that a straight leg lift will be performed and that they should note any difficulty in lifting the leg or if it provokes pain in the back or pelvic region.

The patient is instructed to lift one leg up 20 cm from the surface on beat 1, lower it on beat 2, lift the other leg on beat 3, and lower it on beat 4.

Continue alternating lifting and lowering the legs with each beat. This results in 30 lifts in one minute, corresponding to 15 lifts per leg.

The test is terminated when:

Pelvic tilting occurs in the horizontal plane.

The abdomen pushes forward more than in the basic execution (doming/coning).

Pain occurs in the back and/or pelvis.

The person can no longer continue.

The person cannot maintain the correct tempo.

Number of seconds: ____________________

Was the test terminated due to pain or discomfort? Yes ☐ No ☐

If yes, indicate the location of the pain/discomfort: __________________________________

7. Pelvic Provocation Test (4P Test)

The patient lies on their back. The test-side leg is bent to 90 degrees of hip and knee flexion.

The therapist stabilizes the opposite side at the crest (crista) edge while applying pressure along the length of the femur.

The test is considered positive if it provokes pain in the area around the posterior pelvic joint on the side of the bent leg.

Right Positive Negative

Left Positive Negative
