# Supplementary material for: Impact of Minimal Incision Repair of Rectus Abdominis Diastasis on Quality of Life and Stress Incontinence: A Prospective Study
Source: J Abdom Wall Surg. 2025 Feb 4;3:13830. doi: 10.3389/jaws.2024.13830 (PMC11833112; doi:10.3389/jaws.2024.13830)
Supplement: Supplementary file 2 [file DataSheet3.PDF]

The Disability Rating Index (DRI), Function Index

User Information

The Disability Rating Index (DRI) or Function Index in Swedish was developed by Associate Professor Erik Spangfort, former head of the back section at the orthopedic clinic, Huddinge Hospital, and has been used in clinical contexts since 1980.

The Disability Rating Index is a self-assessed measure of perceived physical disability in patients with musculoskeletal disorders. It encompasses twelve deliberately unspecified daily activities that most people perform or can imagine performing. The questions are arranged in order of increasing physical demands (primarily related to low back pain).

Visual Analog Scales (VAS) have been chosen to enhance the instrument's sensitivity to changes in condition. The instrument has good reliability, validity, and responsiveness (sensitivity to clinically significant changes) (1). The DRI is practical to administer, and 91% of patients complete the instrument entirely correctly.

Over the years, many questionnaires have been modified with good intentions by various users. This counteracts the desirable standardization, which has the significant advantage of allowing comparisons between different studies. Even seemingly marginal modifications result in the loss of the ability to refer to evaluation data for an instrument in the original article.

The evaluation of questionnaires is an ongoing process, and the use of the DRI on the same or other defined patient materials is welcomed.

To maintain the DRI's standard and scientific rigor, we would like to convey the following:

1. The user should not modify, shorten, change scales or calculation methods, alter questions, wording, question order, or translate the DRI. (The DRI will soon be available in a professional Finnish translation.)
2. The user should not reproduce or copy the DRI except for their own use and should not distribute copies to third parties through sale, loan, or other means.
3. The DRI may be revised from time to time. The authors will provide the user with any revised forms. The user shall have the right to continue using an older version of the DRI in connection with ongoing projects or studies where the older version has been used and where the user identifies and marks the forms according to the authors' guidelines.

Åre, 1995-02-08

Bo Salén

Senior Physician

Ref.:

1. Salén BA, Spangfort EV, Nygren ÅL, Nordemar R, **The Disability Rating Index: An instrument for the assessment of disability in clinical settings.** Journal of Clinical Epidemiology 1994; 47(12): 1423-1435.

Function Index (DRI)

(according to Spangfort)

Date: ___________________ Name: __________________________________

Example

Without difficulty **Not at all**

I---I

With some difficulty __With difficulty __With great difficulty

How do you manage the following activities?

Place ONE mark across the line after each question

Please answer ALL the questions

Without difficulty **Not at all**

- Dressing and undressing without help
- Walking ……………………………………….
- Climbing stairs ………………………………….
- Sitting for a long time ……………………….
- Standing bent over a sink ……………
- Carrying a bag or suitcase ……………….
- Making a bed **…………………………………..**
- Running **…………………………………………**
- Light physical work …………………………
- Strenuous physical work …………………..
- Heavy lifting ………………………………………
- Exercise/sports ……………………………….

Calculation of Function Index (Disability Rating Index, DRI)

Measure with a ruler the number of millimeters from the zero point on the left side of the VAS scale ("Without difficulty") to the patient's mark. Measure carefully; do not approximate. Note the result, for example, at the far right of the line. Add the results from the twelve answers and divide by twelve to obtain the mean = DRI (without decimals).

It occasionally happens that patients do not fill in the DRI form as intended. The most serious error is when the patient completely misunderstands the visual analog scale. The most common variations are listed below with guidelines for handling.

1. **The patient does not answer one or more questions.** In principle, the instrument is unreliable in these cases. Even if a mean can be calculated from the answered questions, this mean is affected by which question(s) are omitted due to the activities' relative physical demands. In practical terms, however, we have accepted up to two missed questions and then used the mean of the remaining 11 or 10 answers.
2. **The patient makes two or more marks on the same scale.** In some cases, the patient wants to mark an interval for one or more questions to indicate that the functional impairment varies. Use the mean of the marks on the individual scale as the result.
3. **The patient makes a horizontal line just above the scale to indicate the approximate functional impairment.** Use the midpoint of the mark as the result.
4. **The patient makes a cross or circle on or above the scale.** Use the midpoint of the mark as the result.
5. **The patient writes on the scale "With some difficulty," "With difficulty," "With great difficulty," etc.** Use the following results:
    - Without difficulty: **0**
    - With some difficulty: **25**
    - With difficulty: **50**
    - With great difficulty: **75**
    - Not at all: **100**
