# Supplementary material for: Impact of Minimal Incision Repair of Rectus Abdominis Diastasis on Quality of Life and Stress Incontinence: A Prospective Study
Source: J Abdom Wall Surg. 2025 Feb 4;3:13830. doi: 10.3389/jaws.2024.13830 (PMC11833112; doi:10.3389/jaws.2024.13830)
Supplement: Supplementary file 3 [file DataSheet1.PDF]

Questionnaire Regarding Quality of Life and Urinary Leakage

Name:……………………………….. Date:……………………

Do you experience any of the following problems, and if so, to what extent?

	**Not at all**	**A little**	**Moderate**	**A lot**
	0	1	2	3
1. Needing to go to the toilet very often?	☐	☐	☐	☐
2. Urinary leakage associated with a strong urge to urinate?	☐	☐	☐	☐
3. Urinary leakage associated with physical activity, coughing, or sneezing?	☐	☐	☐	☐
4. Small amounts of urinary leakage (drops)?	☐	☐	☐	☐
5. Difficulty emptying the bladder?	☐	☐	☐	☐
6. Pain or discomfort in the lower abdomen or genital area?	☐	☐	☐	☐

Does urinary leakage and/or prolapse affect you in terms of:

	**Not at all**	**A little**	**Moderately**	**A lot**
	0	1	2	3
1. Ability to perform household chores (cooking, cleaning, washing clothes)?	☐	☐	☐	☐
2. Leisure activities such as walking, swimming, or other physical activities?	☐	☐	☐	☐
3. Entertainment (e.g., movies, concerts)?	☐	☐	☐	☐
4. Ability to travel by car or bus more than 30 minutes from home?	☐	☐	☐	☐
5. Participation in social activities outside the home?	☐	☐	☐	☐
6. Mental health (nervousness, depression, etc.)?	☐	☐	☐	☐
7. Frustration; I feel prevented from doing what I want	☐	☐	☐	☐
